# Women with familial hypercholesterolemia phenotype are undertreated and poorly controlled compared to men

**DOI:** 10.1038/s41598-023-27963-z

**Published:** 2023-01-27

**Authors:** Alberto Zamora, Rafel Ramos, Marc Comas-Cufi, María García-Gil, Ruth Martí-Lluch, Nuria Plana, Lia Alves-Cabratosa, Anna Ponjoan, Celia Rodríguez-Borjabad, Daiana Ibarretxe, Irene Roman-Degano, Jaume Marrugat, Roberto Elosua, Anabel Martín-Urda, Lluis Masana

**Affiliations:** 1 Lipids and Arteriosclerosis Unit, Corporació de Salut del Maresme I la Selva, Girona, Spain; 2 Department of Medical Sciences, School of Medicine, University of Girona, Girona, Spain; 3 Laboratory of Translational Medicine (Translab), School of Medicine, University of Girona, Girona, Spain; 4 Xarxa de Unitats de Lipids de Catalunya (XULA), Girona, Spain; 5 CIBER Cardiovascular Diseases (CIBERCV), Instituto de Salud Carlos III (ISCIII), Madrid, Spain; 6 Institut Universitarid’Investigació en Atenció Primària Jordi Gol (IDIAP Jordi Gol), Maluquer Salvador 11, 170002 Girona, Catalunya Spain; 7 ISV-Girona Research Group, Research Unit in Primary Care, Primary Care Services, Catalan Institute of Health (ICS), Girona, Catalonia Spain; 8 Universitat Rovira I Virgili, Lipids and Arteriosclerosis Research Unit, “Sant Joan” University Hospital, IISPV, CIBERDEM, Reus, Spain; 9 Biomedical Research Institute, (IdIBGi), ICS, Girona, Catalonia Spain; 10 Registre Gironí del Cor Research Group (REGICOR) and Cardiovascular, Epidemiology and Genetics Research Group (EGEC), Hospital del Mar Medical Research Institute (IMIM), Barcelona, Catalonia Spain; 11 Faculty of Medicine, University of Vic-Central University of Catalonia (UVic-UCC), Vic, Spain; 12 Internal Medicine Department, Hospital of Palamos, Serveis de Salut Integrats Baix Empordà, Palamós, Girona, Spain

**Keywords:** Dyslipidaemias, Dyslipidaemias

## Abstract

Familial hypercholesterolemia (FH) is an autosomal dominant disease that has a prevalence of approximately 1/250 inhabitants and is the most frequent cause of early coronary heart disease (CHD). We included 1.343.973 women and 1.210.671 men with at least one LDL-c measurement from the Catalan primary care database. We identified 14.699 subjects with Familial hypercholesterolemia-Phenotype (FH-P) based on LDL-c cut-off points by age (7.033 and 919 women, and 5.088 and 1659 men in primary and secondary prevention, respectively). Lipid lower therapy (LLT), medication possession ratio (MPR) as an indicator of adherence, and number of patients that reached their goal on lipid levels were compared by sex. In primary and secondary prevention, 69% and 54% of women (P = 0.001) and 64% and 51% of men (P = 0.001) were on low-to-moderate-potency LLT. Adherence to LLT was reduced in women older than 55 years, especially in secondary prevention (P = 0.03), where the percentage of women and men with LDL-c > 1.81 mmol/L were 99.9% and 98.9%, respectively (P = 0.001). Women with FH-P are less often treated with high-intensity LLT, less adherent to LLT, and have a lower probability of meeting their LDL-c goals than men, especially in secondary prevention.

## Introduction

Cardiovascular diseases (CVD) are the leading cause of death in women. Although cardiovascular mortality has decreased over the past 40 years, women continue to have poorer cardiovascular outcomes than men, especially at young ages^1^. Women are understudied, underdiagnosed, and undertreated, owing in part to the assumption that they are protected from CVD. Despite the extensive evidence on the well-recognized causal role of LDL-cholesterol (LDL-c) in CVD, women receive less intensive treatment for high LDL-c than men^2^.

Familial Hypercholesterolemia (FH) is an autosomal dominant disease and the most common genetic condition that predisposes individuals to premature development of CVD, with an estimated prevalence of approximately 1/250 individuals^3^. Sixty percent of men and 30% of women under 50 and 60 years old, respectively, with non-treated FH will experience a coronary event. Approximately 20% of the patients with coronary heart disease (CHD) under the age of 55 have FH^4^, a condition that remains widely underdiagnosed and undertreated, and lacks analysis from the perspective of sex or gender. At present, the same FH diagnostic criteria are used for both men and women, without consideration of the variability of LDL-c levels by sex throughout life^5^. Additionally, previous reports indicate that women with FH were received less intensive LLT than men^6^. In this regard, real-world data could a useful tool to examine this point considering the sex perspective and help detect possible inequities^7^.

The study objective was to analyze sex differences regarding LLT in people with FH-phenotype (FH-P) by examining real-world data from more than 2 million patients.

## Methods

The data source for this study was the Information System for the Development of Research in Primary Care (SIDIAP), a clinical database of anonymous longitudinal records that contains information from 6.177.972 patients^8^. It includes diagnoses (International Classification of Diseases [ICD-10]), hospital discharge information (ICD-9/10), laboratory tests, and medications that are dispensed by community pharmacies. The quality of the SIDIAP data for the study of CVD epidemiology has been previously documented^9^.

The authors state that this study complied with the Declaration of Helsinki, and ethical approval was obtained from Primary Health Care Research Jordi Goli Gurina Clinical Research Ethics Committee (Girona) (study code P17/090). The committee is credited by the Institutional Review Board (IRB) (IRB00005101, IRB Organizations (Iorga) (IORG0004303) and Federal Eide Assurance (FWA) for the protection of the human subjects for international (Non-US Institutions-FWA Number 00009235).

Primary Health Care Research Jordi Goli Gurina Clinical Research Ethics Committee (Girona, Catalonia, Spain) approved the exemption of informed consent as this was a retrospective analysis of anonymized databases with information provided by the health authorities. The records were dissociated and contained no personal data; the confidentiality of the participants was guaranteed and the realization of this study involved no risks for them. Its feasibility was dependent on the use of anonymized records and corresponding exemption of informed consent.

We included individuals aged 8 years or older who were alive in December 2014 and had at least one measurement of their LDL-c levels between 2006 and 2014. Exclusion criteria applied to patients with are corded history of hypothyroidism, nephrotic syndrome, or a triglyceride levels ≥ 400 mg/dL at baseline. We considered that participants received LLT if their records contained at least one withdrawal of either statin or ezetimibe prescriptions from the pharmacy office within the 6 months preceding the LDL-c measurement. The missing baseline LDL-c values were calculated using an imputation algorithm, according to the methodology described by Jorgensen et al.^10^ in patients receiving LLT; and using 10 multiple imputations by chained equations in untreated patients. The imputation of pretreated cholesterol levels for participants receiving medications at baseline has been shown to yield estimates that are consistent with reports from randomized clinical trials^11^. The variables included in the imputation models were age, sex, dose and type of LLT, treatment adherence (purchasing record), and the presence of diabetes mellitus (DM). We considered the LDL-c measurement that was closest to December 2014.

Adherence to treatment was calculated according to the "medication possession ratio" (MPR), defined as the proportion of days, within a 6-month period, covered by the lipid-lowering treatment purchased in the pharmacy. Adherence is considered adequate if the MPR values are higher than 80%. LLT treatments were classified according to their ability to reduce LDL-C levels as low, < 30%; moderate, 30–50%; high, 50–60%; and very high, > 60%^12^.

FH-P was defined as untreated LDL-c levels of > 4.91 mmol/L in individuals 8 to 17 years old, > 5.94 mmol/L in individuals 18 to 30 years old, > 6.18 mmol/L in individuals 30 to 39 years old, > 6.95 mmol/L in individuals 40 to 48 years old, and > 6.59 mmol/L in individuals older than 48 years old^13^.

The diagnoses of CVD, which included peripheral arterial disease (PAD), CHD, and ischemic stroke, and the presence of DM, hypertension, hypercholesterolemia and smoking were coded using the ICD-10 and ICD-9 classifications for primary care and hospital records, respectively.

The MEDEA is a socioeconomic deprivation index, validated in the Spanish population. The main components of this index are five socioeconomic indicators recorded by census tract: (a) manual workers: percentage of manual workers aged ≥ 16; (b) unemployment: number of people aged ≥ 16 years and unemployed or actively seeking a job as a percentage of the total economically active population; (c) temporary workers: percentage of people employed in temporary jobs and aged ≥ 16 years; (d) low educational level: percentage of people aged ≥ 16 years with < 5 years of schooling or with no complete basic compulsory education; (e) Low educational level in young people (16–29 years). Higher index values corresponded to greater deprivation, and R refers to the rural population. The MEDEA index has been used to detect possible social inequities by sex in the use of LLT^14^.

### Statistical analysis

Categorical variables were expressed as percentages and continuous variables as means and standard deviations (SDs). Comparisons between groups were measured using generalized linear models: Gaussian, binomial, and multinomial distributions were used for numeric, binary and other categorical variables, respectively. The final significance was obtained using the Rubin's rules for multiple imputations. Statistical analyses were performed using R-software^15^.

## Results

**Figure 1:**
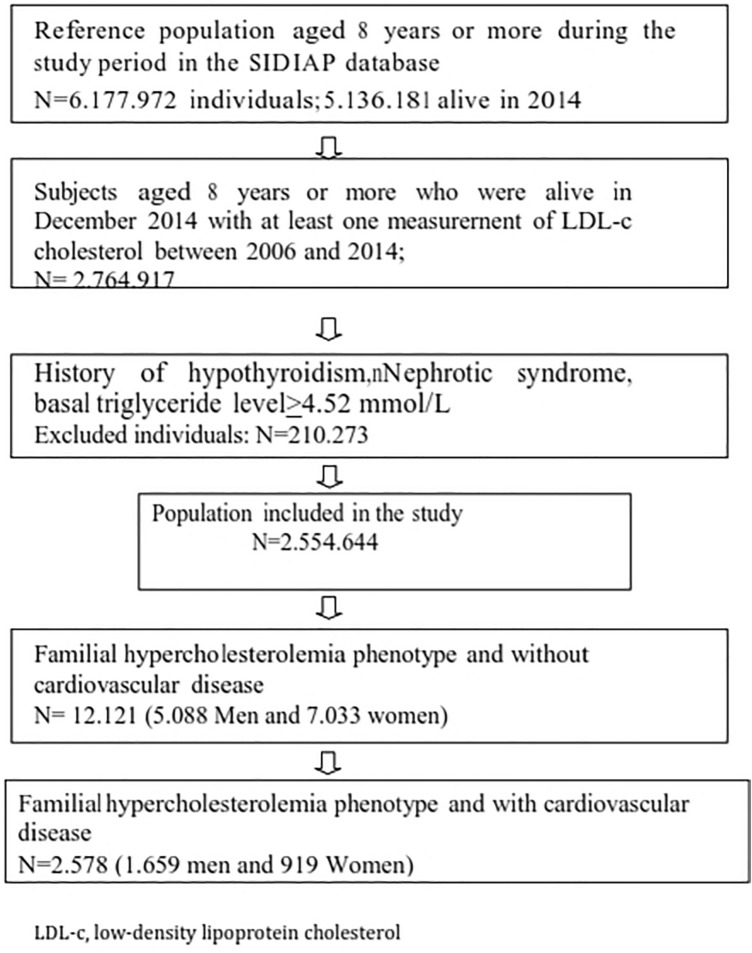
Flow chart summarizing the participant selection in the study. *LDL-c* low-density lipoprotein cholesterol.

**Figure 2:**
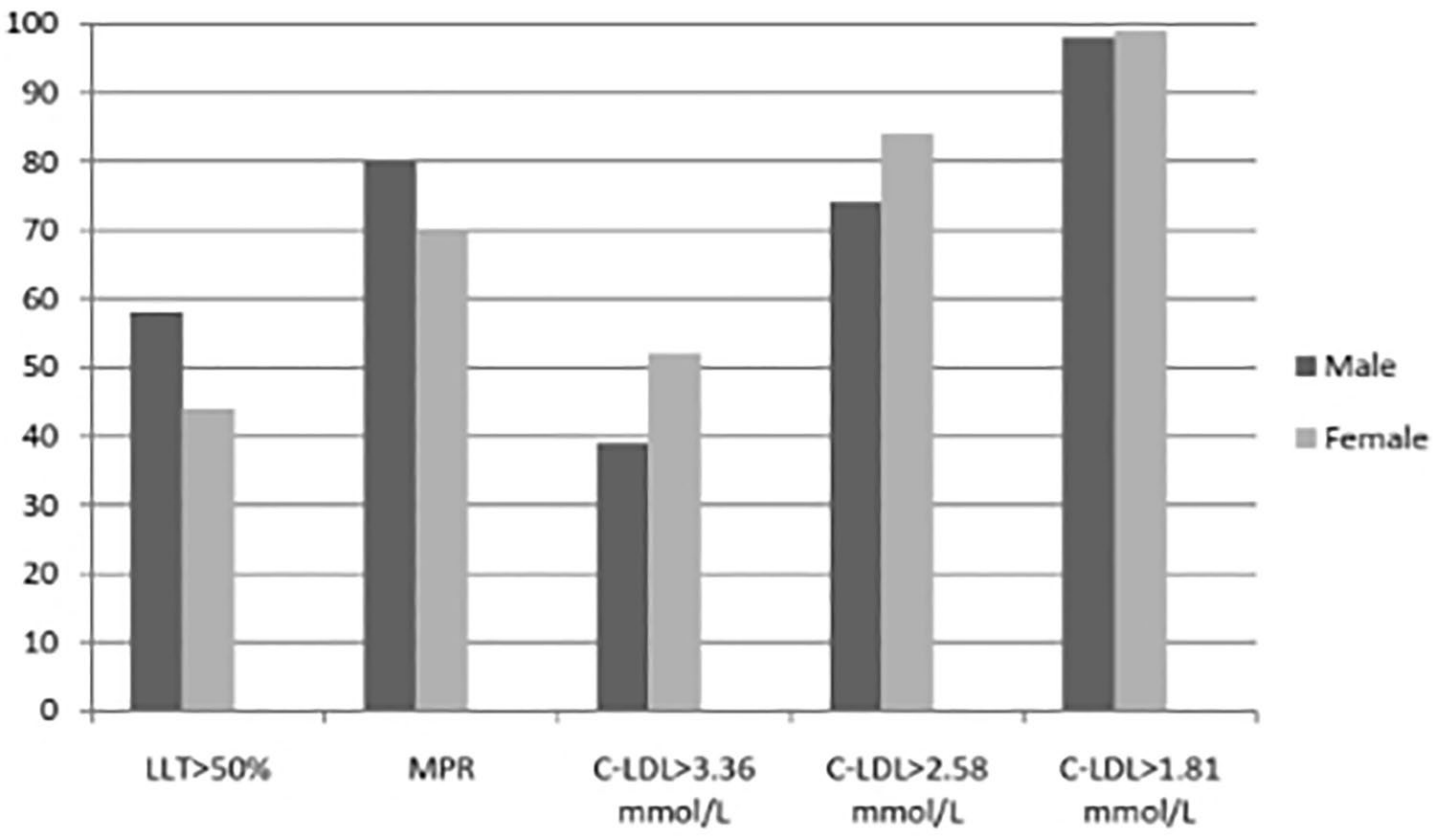
Low-density lipoprotein cholesterol levels by age and sex in treated and untreated participants with Familial Hypercholesterolemia  Phenotype (N = 14.699). *LDL-C* low-density lipoprotein cholesterol (momol/L).

A flowchart summarizing the selection of participants in the study is shown in Fig. 1. At least one LDL-c measurement was recorded between 2006 and 2014 for 2.764.917 individuals. Of these, 1.343.973 women and 1.210.671 men met all the inclusion criteria. A total of 14.699 patients (7.952 women and 6.747 men) fulfilled the criteria for the FH-P definition. LDL-c values in participants treated and untreated with LLT are shown in Fig. 2.

### Primary prevention

Among patients with FH-P, 7,033 women and 5.088 men had no CVD, their characteristics are shown in Table 1. Women were older than men (63 years (15.4) vs 58ears (16), P < 0.001). Baseline LDL-c levels of untreated participants were 7.31 (0.98) mg/dL and 7.34 (0.95) mg/dL in men and women, respectively (P = 0.22). Women showed higher high-density lipoprotein cholesterol (HDL-C) levels than men (1.55 (0.36) mmol/L vs 1.31(0.31) mmol/L, respectively, P = 0.001), lower triglyceride (TG) levels (1.60 (0.71) mmol/L vs 1.72 (0.14) mg/dL, P = 0.001) and lower TG/HDL-c, 1.03 (1.97) vs 1.31(2), P = 0.001).

**Table 1:** Baseline characteristics of population with familial hypercholesterolemia phenotype by sex and the absence–resence of cardiovascular disease.

	FH-P without cardiovascular disease			FH-P with cardiovascular disease		
	Male	Female	P-value	Male	Female	P-value
N (%)	5.088 (42)	7.033 (58)		1.659 (64)	919 (36)	
Age, mean (SD)	58 (16)	63 (15)	0.001	67 (11)	73 (11)	0.001
Diabetes mellitus (%)	866 (17)	1.135 (16)	0.37	580 (35)	319 (35)	0.90
Hypertension (%)	2.070 (41)	3.394 (48)	0.001	1.182 (71)	741 (81)	0.001
Current smoker (%)	1.551 (30.5)	1.160 (16.5)	0.001	423 (25)	86 (9)	0.001
BMI, mean (SD)	28 (4)	28 (5)	0.63	29 (4)	30 (5)	0.01
Untreated LDL-C, mmol/l mean (SD)	7.31 (0.98)	7.34 (0.95)	0.22	7.44 (0.90)	7.44 (0.93)	0.96
LDL-C, mmol/L mean (SD) (on LLT)	4.62 (1.5)	4.65 (1.60)	0.14	3.41(1.24)	3.77 (1.29)	0.001
HDL-C, mmol/L mean (SD)	1.31 (0.31)	1.55 (0.36)	0.001	1.24 (0.28)	1.44 (0.33)	0.001
TG, mmol/L mean (SD)	1.72 (0.14)	1.6 (0.71)	0.001	1.65 (0.4)	1.69 (0.73)	0.21
TG/HDL-C (SD)	1.31 (2)	1.03 (1.97)	0.001	1.33(1.42)	1.17 (2.21)	0.002
Basal glucosa mmol/L (SD)	5.8 (1.8)	5.7 (1.8)	0.003	6.4 (2.4)	6.2 (2.3)	0.13
HBA1c, % (SD)	6 (1)	6.1	0.37	6 (1)	6 (1)	0.89
Creatinine mmol/L mean (SD)	0.09 (0.04)	0.05 (0.03)	0.001	0.09 (0.04)	0.08 (0.04)	0.001
SBP mmHg, mean (SD)	131 (14)	129 (16)	0.001	132 (15)	133 (17)	0.03
DBP mmHg, mean (SD)	78 (10)	75 (10)	0.001	74 (10)	74 (10)	0,16
MEDEA (Q1), N (%)	290 (9)	466 (11)	0.05	83 (10)	56 (11)	0.66
MEDEA (Q2), N (%)	485 (15)	708 (16)	0.35	129 (15)	84(16)	0.69
MEDEA (Q3), N (%)	610 (19)	862 (20)	0.61	170 (20)	96 (18)	0.60
MEDEA (Q4), N (%)	592(19)	816 (19)	0.97	150 (18)	92 (18)	0.98
MEDEA (Q5), N (%)	542 (17)	714 (16)	0.46	164 (19)	101 (19)	0.96
Rural population, N (%)	664 (21)	812(18)	0.02	153 (18)	92(18)	0.90

*BMI* body mass index, *LDL-C* low-density lipoprotein cholesterol, *LLT* Lipid lowering therapy, *HDL-C* high-density lipoprotein cholesterol, *TG* triglycerides, *HBA1c* glycosylated hemoglobin, *SBP* systolic blood pressure, *DBP* diastolic blood pressure, *SD* standard deviation, *MEDEA* socioeconomic deprivation index.

Among patients with FH-P, 14% of women and 19% of men were not receiving LLT (P = 0.001), 69% of women and 64% of men were on low-to-moderate LLT (P = 0.001), and low use of statins plus ezetimibe was observed in both sexes: 3.2% and 3.6% in women and men, respectively (P = 0.2). The most used statin was simvastatin 20 mg (35.6% and 33% in women and men, respectively). LDL-C levels in persons receiving LLT were 4.65 (1.61) mmol/L and 4.60 (1.49) mmol/L in women and men, respectively (P = 0.10). Approximately 84% of women and 75% of men with FH-P and no CVD had LDL-C values above 2.5 mmol/L (P = 0.001) (Table 2).

**Table 2:** Characteristics of lipid-lowering therapy in Familial Hypercholesterolemia Phenotype population.

	FH-P without cardiovascular disease			FH-P with cardiovascular disease		
	Male N = 5.088	Female N = 7.033	P-value	Male N = 1659	Female N = 919	P-value
Absence of treatment, N, %	969 (19)	977 (14)	0.001	49 (3)	21 (2)	0.35
Low LLT, N, %	442 (9)	781 (11)	0.001	57 (3)	56 (6)	0.001
Moderate LLT; N, %	2.785 (55)	4.099 (58)		628 (38)	438 (48)	
High LLT, N, %	833 (16)	1087 (15)		833 (52)	369 (40)	
Very High LLT, N%	59 (1)	89 (1)		92 (6)	36 (4)	
Statins and ezetimibe; N, %	184 (4)	222 (3)	0.19	196 (12)	86 (9)	0.07
Statin pattern dose^a^, N, %	Simvastatin 20 1.353 (33)	Simvastatin 20 2.140(36)	0.003	Atorvastatin 40. 555 (34)	Atorvastatin 40. 252 (28)	0.001
	Simvastatin 40. 784 (19)	Simvastatin 40 1.114 (18)		Simvastatin 20 189 (12)	Simvastatin 20 168 (19)	
	Atorvastatin 40. 607 (15)	Atorvastatin 40 825 (14)		Atorvastatin 80 237 (15)	Atorvastatin 80 99 (11)	
Adherence (MPR)	0.6 (0.4)	0.6 (0.3)	0.001	0.8 (0.3)	0.7 (0.3)	0.003
Patients with LDL-C > 3.36 mmol/L, N, %	3.612 (71)	5.275 (75)	0.001	647 (39)	478 (52)	0.001
Patients with LDL-C > 2.5 mmol/L; N; %	4.731 (93)	6.681 (95)	0.001	1.228 (74)	772 (84)	0.001
Patients with LDL-C > 1.81 mmol/L mg/dL; N; %	5.037 (99)	6.963 (99)	0.1	1.626 (98)	910 (99)	0.001

*FH-P* familial hypercholesterolemia phenotype, *LLT* Lipid lowering therapy, *MPR* medication possession ratio, *LDL-C* low-density lipoprotein cholesterol.

^a^Three most used statins.

### Secondary prevention

Cardiovascular disease was present in 24.6% of men and 11.5% of women with FH-P. Women were older than men (73 vs 67 years old, respectively; (P = 0.001). The age of presentation of the first CVD event was older in women than in men (66.2 years old vs 58.6 years old, respectively (p < 0.00001). The basal LDL-c levels in persons without LLT were 7.44 (0.93) mmol/L and 7.44 (0.90) mmol/L in women and men, respectively (P = 0.96). In secondary prevention, women showed higher HDL-c levels compared to men (1.44 (0.33) mmol/L vs 1.24 (0.28) mmol/dL, respectively, P = 0.001) (Tables 1 and 2).

**Figure 3:**
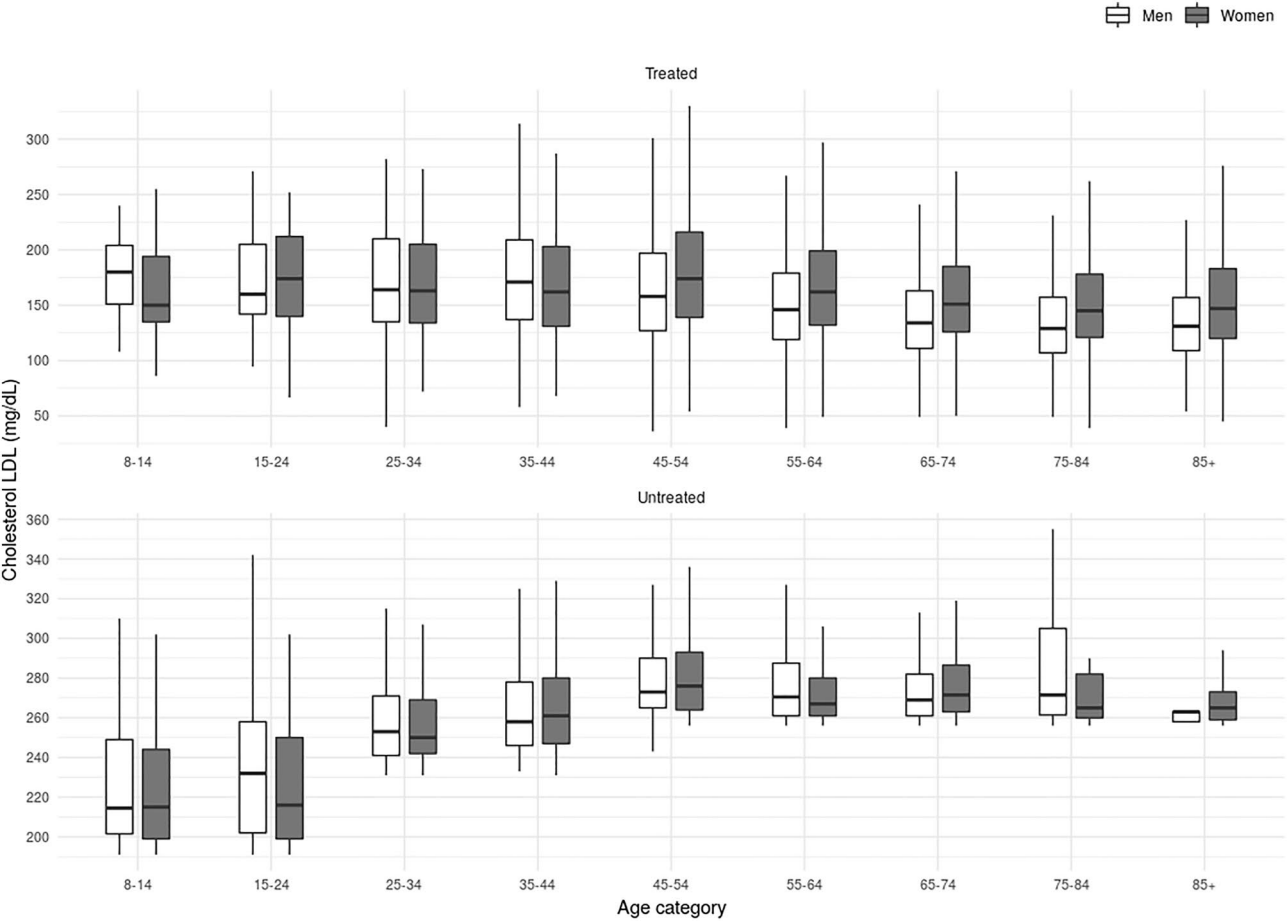
Characteristics of lipid lowering treatment potency, adherence, and LDL-C goal achievement in familial hypercholesterolemia phenotype population in secondary prevention. *LLT* Lipid Lowering therapy, *LLT potency ≥ 50* Lipid lowering therapy with low-density lipoprotein cholesterol reduction capacity ≥ 50%, *MPR* medication possession ratio, *LDL-C* low-density lipoprotein cholesterol.

Only 44% of women compared to 58% of men were on LLT of high or very high potency (P = 0.001) (Table 2). Statin plus ezetimibe was used in 9% and 12% of women and men, respectively (P = 0.07). The most used statin was atorvastatin 40 mg (28% and 34% in women and men, respectively) (P = 0.001). LDL-C levels in persons receiving LLT were 3.77 (1.29) mmol/L and 3.41 (1.23) mmol/L in women and men, respectively (P ≤ 0.001). The percentages of patients with FH-P and CVD who reached LDL-C levels < 1.81 mmol/l were 1% vs 2of females and males, respectively (P = 0.001) (Fig. 3).

### LLT potency and adherence (MPR)

The population with FH-P presented no differences by sex regarding the potency of the received LLT until the age of 35; among 35 to 85 year-olds, women received LLT of lower potency.

Adherence to LLT by MPR was also lower in ≥ 55-year-old women (Table 3).

**Table 3:** Lipid-lowering therapy and adherence (medication possession ratio) by sex and age.

Age (years)	LLT	Women, %	Men, %	P-value
8–14	Low-moderate potency	11.8 (14.7%)	10.4 (13.1%)	0.940
	High-very high potency	1.7 (2.1%)	1.0 (1.3%)	
	Without LLT	67.0 (83.2%)	68.0 (85.6%)	
	MPR	38.8% (35.4%)	50.9% (32.2%)	0.444
15–24	Low-moderate potency	26.5 (23.0%)	17.4 (19.0%)	0.558
	High-very high potency	5.7 (4.9%)	8.3 (9.1%)	
	Without LLT	83.0 (72.0%)	66.0 (72.0%)	
	MPR	49.5% (28.8%)	47.1% (28.4%)	0.681
25–34	Low-moderate potency	60.1 (29.9%)	76.5 (30.7%)	0.848
	High-very high potency	24.2 (12.0%)	34.8 (14.0%)	
	Without LLT	117.0 (58.1%)	138.0 (55.4%)	
	MPR	54.5% (26.9%)	49.9% (27.4%)	0.302
35–44	Low-moderate potency	148.4 (37.9%)	381.5 (46.0%)	0.005
	High-very high potency	53.9 (13.8%)	132.1 (15.9%)	
	Without LLT	189.0 (48.3%)	315.0 (38.0%)	
	MPR	57.6% (27.7%)	55.4% (27.8%)	0.420
45–54	Low-moderate potency	498.8 (61.2%)	688.3 (57.0%)	0.058
	High-very high potency	165.6 (20.3%)	307.3 (25.5%)	
	Without LLT	151.0 (18.5%)	211.0 (17.5%)	
	MPR	61.1% (28.1%)	64.9% (27.6%)	0.068
55–64	Low-moderate potency	1417.0 (68.9%)	1091.6 (63.8%)	0.000
	High-very high potency	426.8 (20.8%)	495.0 (28.9%)	
	Without LLT	213.0 (10.4%)	124.0 (7.2%)	
	MPR	66.5% (27.5%)	71.7% (26.8%)	0.000
65–74	Low-moderate potency	1705.3 (74.5%)	1022.7 (63.3%)	0.000
	High-very high potency	488.2 (21.3%)	544.7 (33.7%)	
	Without LLT	96.0 (4.2%)	49.0 (3.0%)	
	MPR	73.0% (26.2%)	78.4% (24.1%)	0.000
75–84	Low-moderate potency	1121.6 (75.0%)	520.1 (65.6%)	0.002
	High-very high potency	328.0 (21.9%)	250.6 (31.6%)	
	Without LLT	45.0 (3.0%)	22.0 (2.8%)	
	MPR	76.0% (24.7%)	80.0% (23.1%)	0.033
85 +	Low-moderate potency	384.3 (77.8%)	103.8 (69.4%)	0.139
	High-very high potency	86.5 (17.5%)	42.7 (28.6%)	
	Without LLT	23.0 (4.7%)	3.0 (2.0%)	
	MPR	73.5% (26.5%)	77.5% (24.9%)	0.362

*LLT* Lipid lowering therapy, *MPR* medication possession ratio, *LDL-C* low-density lipoprotein cholesterol.

In the multivariate analysis of participants in both primary and secondary prevention, women had lower odds of receiving LLT with a LDL-c lowering potency of at least 50% (Table 4). In secondary prevention, female sex was also associated with a lower intensity of the received LLT.

**Table 4:** Multivariate analysis in primary and secondary prevention.

Level	OR (IC 2.5–97.5%)	P value
Primary prevention		
High intensity LLT	1.008 (0.98–1.03)	0.50
LDL-C reduction by at least 50%	1.082 (1.05–1.11)	0.00001
LDL-C < 3.36 mmol/L	1.07 (1.04–1.1)	0.00001
LDL-C < 2.58 mmol/L	1.029 (1.01–10.4)	0.0003
LDL-C < 1.81 mmol/L	1.003 (1–1.006)	0.05
Secondary prevention		
High intensity LLT	1.09 (1.04–1.15)	0.0005
LDL-C reduction by at least 50%	1.17 (1.11–1.23)	0.00001
LDL-C < 3,36 mmol/L	1.17 (1.11–1.23)	0.00001
LDL-C < 2.58 mmol/L	1.10 (1.05–1.16)	0.0002
LDL-C < 1.81 mmol/L	1.007 (0.99–1.02)	0.36

Primary prevention adjusted by age, high blood pressure, smoking and body mass index. Secondary prevention adjusted by age, high blood pressure, smoking.

*LDL-c* low-density lipoprotein cholesterol, *LLT* Lipid lowering therapy.

### Deprivation rate

**Figure 4:**
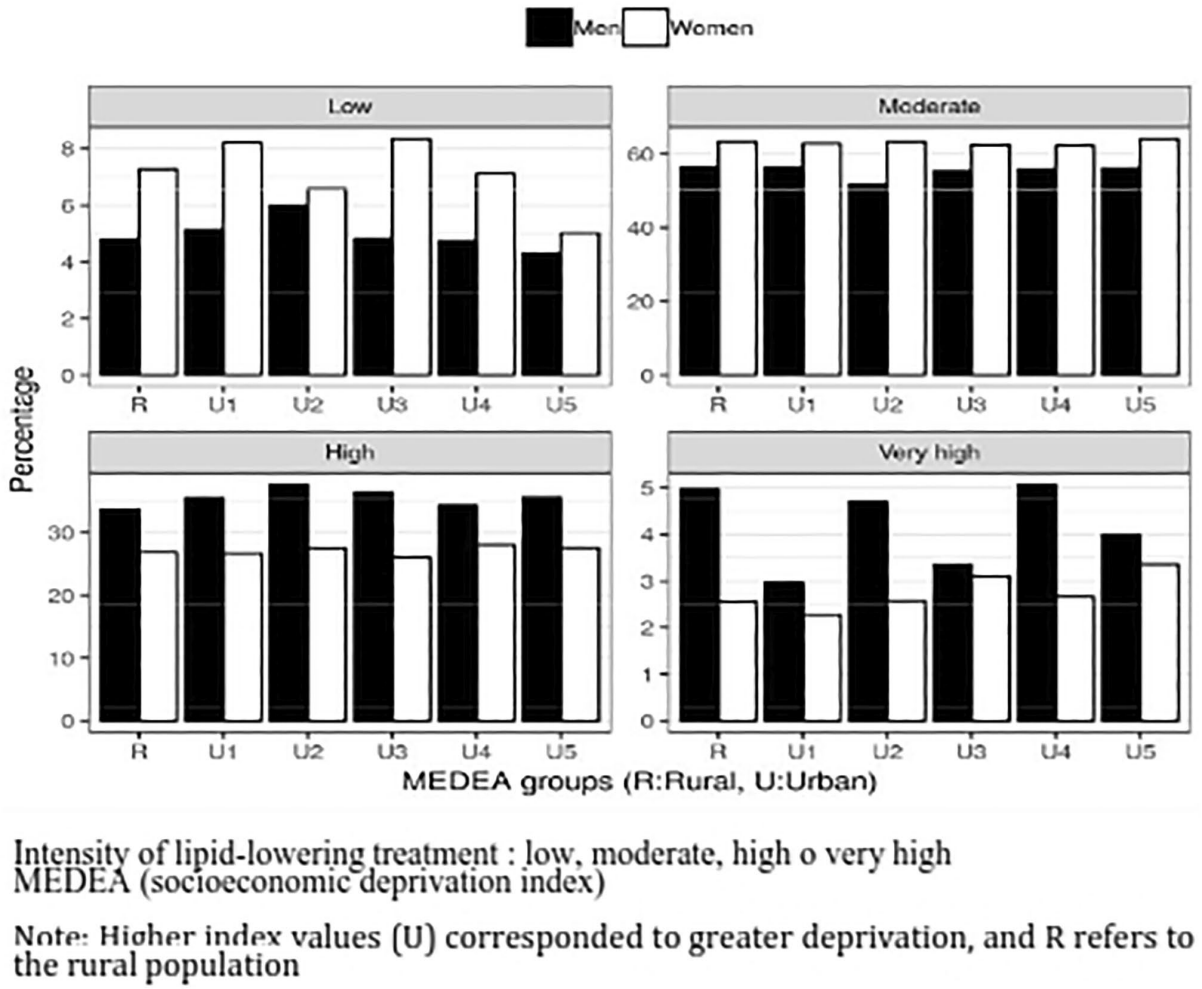
Intensity of lipid-lowering treatment by sex and MEDEA (socioeconomic deprivation index). Intensity of lipid-lowering treatment: low, moderate, high, very high. MEDEA (socioeconomic deprivation index). Higher index values (U) corresponded to greater deprivation and R refers to the rural population.

No sex differences in the intensity of LLT were observed by the MEDEA deprivation index. The highest LDL-C levels were observed in women in the lowest category (U1). The percentage of women treated with LLT of high intensity was lower than that of men regardless of the MEDEA index (Fig. 4).

### Multivariate analysis

Multivariate analysis by sex in primary and secondary prevention confirmed the lower intensity of LLT in women in secondary prevention and the lower proportion of women that achieved lipid goals in primary and secondary prevention (Table 4).

## Discussion

The analysis of real-world data of persons with FH-P showed important sex differences regarding its management and treatment that should be taken into consideration. Despite the evidence on the beneficial use and safety of statin treatment in both sexes, a lower proportion of women received high-intensity LLT compared to men in this FH-P population, especially in secondary prevention. The percentages of patients who reached their goals on LDL-C levels were small, especially in women: 95% and 99% of women in primary and secondary prevention, respectively, did not attain it. Women also presented lower adherence to LLT.

We observed a different distribution of CVRF by sex in patients with FH-P. Women were older, had a higher prevalence of hypertension and a lower prevalence of smoking habit in both primary and secondary prevention. Women had higher BMI values than men only in secondary prevention. The prevalence of DM was very similar in both sexes in primary and secondary prevention. In the general population the sex differences regarding CVRF prevalence are notable^16^. But surprisingly, there is little information regarding the distribution of CVRF by sex in the population with FH-P, which also showed variation by sex^17^. Cardiovascular risk factors have a different impact on CVD risk in women and men with FH^18^, and thus clinical data by sex is needed in the population with FH-P.

Evidence indicates that statins are equally effective in both men and women in the prevention of CVD in high-risk populations. The Cholesterol Treatment Trialists' Collaboration study observed that LLT in women and men at similar baseline CVD risk had comparable relative reductions in risk^19^. In the population with FH, LLT is recommended to reduce the risk of CVD, without differences according to sex^6^. In our study, this treatment and its control were suboptimal in both, men and women. Women received more LLT in primary prevention but, oddly, were treated less intensively than men. Even more, this lower intensity LLT was especially evident in women in secondary prevention. The European Atherosclerosis Society Familial Hypercholesterolaemia Studies Collaboration (FHSC) global registry, which includes 61.612 individuals, showed greater use of more potent lipid-lowering medication in men than in women^20^.

A recent prespecified analysis of IMPROVE-IT demonstrated a greater relative risk reduction in CVD incidence with the use of ezetimibe/simvastatin than with placebo/simvastatin in women^21^. In the present study, we observed scarce use of combination therapy in both sexes, especially in women.

In our population, LDL-C levels in persons without LLT were similar in both sexes. Among treated participants, however, LDL-C levels were significantly higher in women due to lower use of high-intensity LLT, especially in secondary prevention (52% of women vs. 39% of men had LDL-C values > 3.36 mmol/L). In line with this, the CASCADE-FH study reported that women with FH were 40% less likely to receive statin treatment, 32% less likely to achieve the recommended target LDL-c level, and 21% less likely to achieve a 50% decrease in LDL-C levels compared to men with FH^22^.

Recent data have shown similar cardiovascular benefits in both sexes using plasma proprotein convertase subtilisin/kexin type 9 inhibitors (iPCSK9)^23^, although the LIPID-REAL Registry^24^ has shown lower LDL-c reduction in women in the 2 highest quartiles. Nevertheless, the study on the use of iPCSK9 in Catalonia (Spain) from the Catalan Department of Health observed that women only received 41% of a total of 1.917 provided treatments^25^.

Previous analysis reported higher levels of LDL-C in younger women of lower social classes^26^. Our study showed the highest levels of LDL-C in women with the lowest socioeconomic deprivation rate, but the lower intensity of the LLT in women was independent of their socioeconomic status.

We detected a lower adherence to LLT in women older than 55 years of age compared to men of the same age group. In a recent meta-analysis of 3.022 potential publications, women presented a 10% increase in the odds of non-adherence to statin treatment compared to men^27^. Another analysis of more than 400.000 statin users showed an association of the following factors with non-adherence: female sex, primary prevention, and treatments with simvastatin, lovastatin, pravastatin, and fluvastatin^28^. The reasons for these lower rates of adherence to statins in women may include) pregnancy planning that prevent them from initiating or stopped LLT. We also observed a lower adherence in women in peri- and post-menopausal age ranges, both in primary and secondary prevention. Women and clinicians may no have the same point of view when prioritizing prevention of cardiovascular disease because of a misconceived lower risk in women. Alternatively, women frequently serve as caregivers for family members and caregivers often have lower rates of medication adherence^29^. Recent studies have indicated that women may have a higher incidence of adverse effects due to treatments with statins, especially myalgia and onset of diabetes, which may also result in lower adherence^30^. Women have a higher activity of cytochrome P450 3A4, so they would have a greater capacity to metabolize lipophilic statins than men and also a higher susceptibility to present interactions^31^. Women with CVD have more comorbidities, a higher prevalence of polypharmacy and a higher risk of pharmacological interactions than men^32^. However, in a recent meta-analysis that assessed the efficacy and safety of statins, women represented only 27% of the included population^19^. In our study, the most prescribed statin-type in both sexes were simvastatin and atorvastatin in primary and secondary prevention, respectively, both of these statins are lipophilic. Currently, there are no recommendations for the prescription of LLT specific by sex^6^.

Women between 25 and 50 years of age are less likely to be diagnosed with FH, to have an assessment of the high Dutch Lipid Clinic Network Score, and to receive a recommendation for a genetic study because diagnostic criteria regarding lipid values are currently used without consideration of the physiological differences between sexes^33^. In a previous study based on real-world data, we observed a lower prevalence of FH-P in women from 25 to 55 years old, despite a higher number of women with LDL-C determinations, more frequent use of the health services by women and higher mortality associated to FH in older men^34^.

It appears that FH eliminates the “female advantage” against CHD that healthy women enjoy during their premenopausal years. A lower prevalence of FH diagnosis, whether clinical or genetic, may lead to a lower risk perception in physicians and patients, especially in young women, with possible impact on the intensity of treatment and adherence to it. In population with FH o FH-P, risk functions stratified by sex and age are necessary. If we want to improve early detection, treatment, and adherence in women and men with FH-P, it is important to emphasize the priority of describing LDL-c cut-off points by age, sex, country, and ethnicity.

Even though women with FH-P received less intensive LLT, they had lower adherence to it, and reached their goal LDL-c values less frequently, their CVD prevalence was lower and the increase in the incidence of CVD occurred at older age than men. This suggests that other cardio-protective factors, like hormone factors, higher HDL-C levels, lower prevalence of other CVRF, like tobacco or higher levels of triglycerides/high-density lipoprotein cholesterol in men might play a role. It is important to determine whether the same LDL-C value has the same impact on cardiovascular risk in women and men in different populations^35^. Recent findings have evidenced a sex-specific differential causal effect of genetically increased LDL-C on the risk of CVD. These observations implicate that women are less susceptible to LDL-C-associated CVD compared to men and male patients might benefit more from LDL-C targeted therapies than female patients^36^. Sex‐differential effects should be taken into account in the design of clinical trials. If we want to move towards personalized medicine, we must begin by considering differences by sex.

### Strengths and limitations of the study

SIDIAP includes medical data from 85% of the Catalan population; it has a substantial potential to provide a global vision on the FH-P epidemiology. Our study population included all individuals who had an LDL-C test during 10 years, which was 53.8% of the general population older than 7 years and 68.2% of persons older than 45 years; thus, we must acknowledge some selection bias, especially at younger ages.

The main limitation of our study was the FH diagnostic method, based on the FH phenotype, which could contribute to overestimate the diagnosis, especially due to inclusion of persons with polygenic familial hypercholesterolemia. The cut-off points used for LDL-C have a sensitivity of 91%, a specificity of 71% and a positive predictive value of 74% for genetically defined HF^13^. However, a concept of Familial Hypercholesterolemic syndrome (which includes heterozygous familial hypercholesterolemia, homozygous familial hypercholesterolemia, polygenic familial hypercholesterolemia, and familial hypercholesterolemia combined with hypertriglyceridemia) has been recently defined on the basis that all these types of hypercholesterolemia present a clinically relevant excess of CVD^37^. From 2014 to date, the percentage of people receiving LLT has improved, although recent data indicate that there is scope for improvement^22^. Finally, another limitation is the lack of availability of information in SIDIAP regarding family history, Lp (a) levels and use of iPCSK9 or other cardiovascular medications in addition to LLT.

In conclusion, unexplained sex differences were detected in the use of high-intensity LLT, which was decreased in women with FH-P. This may lead to worsened lipid control and lower achievement of LDL-c goals in women compared to men, especially in secondary prevention. There were differences by sex regarding adherence to treatment, which require further research.

## Data Availability

The datasets analysed in this study are not publicly available due to legal reasons related to data privacy protection but are available from the corresponding author on reasonable request.
